# Large‐Scale Clustered Transcriptional Silencing Associated With Cellular Senescence

**DOI:** 10.1111/acel.70015

**Published:** 2025-02-19

**Authors:** Aditi U. Gurkar, Satoshi Okawa, Christelle Guillermier, Kritika Chaddha, Matthew L. Steinhauser

**Affiliations:** ^1^ Aging Institute of UPMC and the University of Pittsburgh School of Medicine Pittsburgh Pennsylvania USA; ^2^ Division of Geriatric Medicine, Department of Medicine University of Pittsburgh School of Medicine Pittsburgh Pennsylvania USA; ^3^ Center for Pulmonary Vascular Biology and Medicine, Pittsburgh Heart, Lung, and Blood Vascular Medicine Institute, Division of Cardiology, Department of Medicine University of Pittsburgh School of Medicine and UPMC Pittsburgh Pennsylvania USA; ^4^ Department of Computational and Systems Biology University of Pittsburgh School of Medicine Pittsburgh Pennsylvania USA; ^5^ McGowan Institute for Regenerative Medicine University of Pittsburgh School of Medicine Pittsburgh Pennsylvania USA; ^6^ Center for Nanoimaging, Division of Genetics Department of Medicine, Brigham and Women's Hospital Cambridge Massachusetts USA; ^7^ Division of Cardiovascular Medicine, Department of Medicine University of Pittsburgh School of Medicine Pittsburgh Pennsylvania USA

**Keywords:** aging, cellular senescence, imaging mass spectrometry, scRNA‐seq, transcription

## Abstract

Senescence is a cell fate associated with age‐related pathologies; however, senescence markers are not well‐defined. Using single cell multi‐isotope imaging mass spectrometry (MIMS), we identified hypercondensed, transcriptionally silent DNA globules in a senescence model induced by dysfunctional telomeres. This architectural phenomenon was associated with geographically clustered transcriptional repression across somatic chromosomes with over‐representation of cell cycle genes. Senescence‐stimuli was associated with a higher frequency of cells that exhibited geographically concentrated transcriptional repression relative to control cells. This phenomenon was also observed in multiple other senescence models, including replicative senescence and irradiation. We further identified an enrichment of common pathways in all models of senescence, suggesting a common cellular response to this silencing phenomenon. Such large‐scale clustered silencing of chromosomal segments rather than individual genes may explain senescence heterogeneity and a putative trajectory toward deep, irreversible senescence.

## Graphical abstract

Senescence inducing stimuli leads to large‐cluster hypercondensed DNA globules. This architectural phenomenon is associated with geographically clustered transcriptional repression. Such large‐scale clustered silencing of chromosomal segments may drive deep senescence through two possible ways: (1) one or more critical combination of genes, for example cell cycle genes, are stochastically sequestered in dense DNA globules (2) activation of a cellular stress program that in turn drives senescence independent of the specific genes.

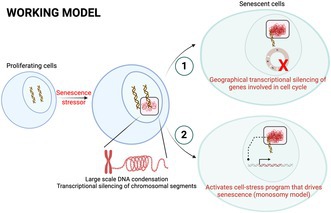

1

Senescence is a hallmark of biological aging, with growing evidence that accumulation of senescent cells contributes to multiple age‐related diseases (Gorgoulis et al. 2019). Senescent cells display complex morphological and heterogeneous molecular changes that differ across cell‐types and triggers, and as such there are no universal molecular markers (Suryadevara et al. 2024). The broad range and heterogeneity of senescence phenotypes—*senotypes*—provides rationale for the integration of multi‐modality single cell analytic tools to better define senescence (Gurkar et al. 2023).

Multi‐isotope imaging mass spectrometry (MIMS) is a method of nanoscale quantitative imaging of stable isotope tracers (Gyngard and Steinhauser 2019; Steinhauser et al. 2012). Here, we applied MIMS to quantitatively map newly synthesized RNA in a genetic model of cellular senescence, whereby telomere dysfunction is induced by loss of *telomeric repeat binding factor 2* (*Trf2*) upon tamoxifen exposure in mouse embryonic fibroblasts (MEFs) (Alder et al. 2015) (Figure S1). *Tfr2* deletion leads to telomere uncapping resulting in rapid and uniform senescence. With MIMS, single mass images reveal complementary cellular and organellar details, including cellular and nuclear contours (CN^−^ or S^−^ images) and phosphorus rich chromatin (P^−^ images); whereas hue saturation intensity isotopic ratio images provide quantitative maps of stable isotope labeling (Figure 1A and Figure S2a) (Guillermier et al. 2017; Steinhauser et al. 2012). We performed saturating labeling of DNA over successive passages with ^13^C‐thymidine prior to and during temporally defined induction of senescence by *Trf2* loss of function (Figure 1A). Hence, an increase in ^13^C signal within any given intranuclear domain indicates a higher DNA concentration relative to regions with lower ^13^C (Guillermier et al. 2024). We then administered ^15^N‐uridine pulse (120 min) and chase (120 min) in experimental (*Trf2* ^*−/−*^) and control (*Trf2* ^*+/+*^) cells. A visually striking feature of the senescence model was the emergence of globular ^31^P‐rich structures that were intensely ^13^C‐thymidine‐labeled, consistent with hypercondensed DNA (Figure 1B). PCA analyses of intranuclear pixel values, using the C and N isotopic ratio measurements and the direct ^12^C^14^N, ^31^P, and ^32^S ion counts as input, demonstrated colocalization of condensed DNA globules in the interior of the nucleus with condensed DNA from the nuclear lamina, a region known to exhibit heterochromatin condensation and transcriptional silencing, suggesting similar elemental composition of these two domains (Figure 1C). These globules exhibited dimensions up to ~1 μm in diameter (Figure 1D,E). Indeed, *Trf2* ^*−/−*^ cells exhibited a general increase in both size and frequency of transcriptionally silent regions of condensed DNA (Figure S2b). The change in DNA condensation was also reflected by metrics of intranuclear ^13^C‐thymidine labeling heterogeneity (Figure 1F). Coincident with remodeling of the DNA architecture, there was a global reduction in nuclear RNA synthesis (Figure 1G) driven by intranuclear DNA foci that were remarkably devoid of ^15^N‐uridine labeling. Despite persistence of ^15^N‐uridine labeled RNA in intranuclear compartments such as nucleoli after 120 min label‐free chase, hypercondensed DNA globules continued to be impervious to ^15^N‐uridine labeling (Figure S2).

**FIGURE 1:**
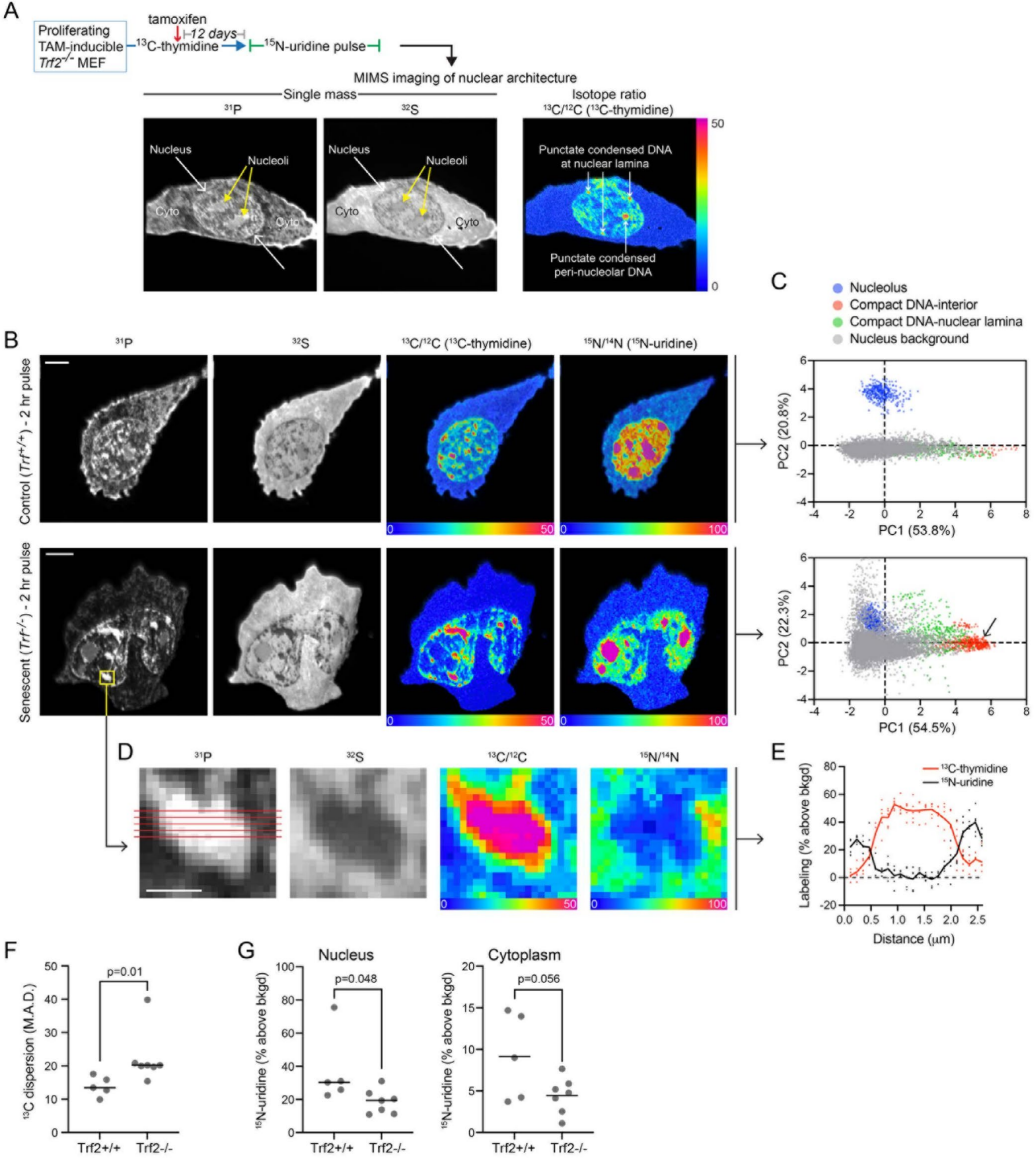
Cellular senescence drives remodeling of the DNA architecture and transcriptional silencing. (A) Schematic of tamoxifen inducible genetic senescence model and multi‐isotope imaging mass spectrometry (MIMS) imaging of nuclear architectural features. Single mass images show intracellular and organellar details. Hue saturation intensity isotope ratio images quantitatively map stable isotope tracer incorporation with the low end of the scale (blue) set to natural background and the top end of the scale set to reveal differences in label incorporation (expressed as % above background). Cyto = cytoplasm. (B) Representative control and senescent cells after 120 min ^15^N‐uridine pulse. Scale bars = 5 μm. (C) PCA plot of intranuclear pixels from (B) using mass quantification and isotope ratio measurements as input. Intranuclear DNA globules formed a discrete subpopulation (red dots, arrow) that overlapped with the condensed DNA at the nuclear lamina (green dots). (D) Punctate area of highly concentrated DNA, devoid of nascent ^15^N‐labeled RNA. Scale bar = 1 μm. (E) Line profiles from D capturing the paucity of ^15^N‐uridine labeling at the center of the condensate. (F) Metric of dispersion (M.A.D.) demonstrates increased heterogeneity of the DNA distribution in senescent cells. (*n* = 5 control; *n* = 7 *Trf2* ^−/−^ cells); Mann–Whitney test performed due to non‐normal distribution (Shapiro–Wilk). (G) Reduction in nuclear nascent RNA labeling in senescent cells (*n* = 5 control; *n* = 7 *Trf2* ^−/−^ cells); two‐tailed *t*‐test performed for cytoplasm; Mann–Whitney test performed for nucleus due to non‐normal distribution (Shapiro–Wilk).

Given the spatial dimensions of the observed DNA dense and transcriptionally silent globules (Figure 1B, inset), we reasoned that associated transcriptional effects operate on a scale larger than any individual gene—that is, repression of chromosomal segments. Any gene contained within a chromosomal segment forming such a DNA globule should exhibit hemi‐allelic silencing. We reasoned that integration of single cell transcriptomics (scRNA‐seq) would allow testing of specific predictions implied by this model. We performed scRNA‐seq in the *Trf2* loss of function, senescence model to test our a priori prediction that putative senescent cells would exhibit increased geographic clustering of repressed genes. UMAP visualization demonstrated emergence of *Trf2* ^*−/−*^ cells that were distinct from controls with down regulation of cell cycle pathways (Figure 2A; Figure S4; Tables S1 and S2). For the purposes of modeling hemi‐silencing, we defined a silenced gene as one exhibiting a ≥ 50% reduction in transcript counts relative to the mean of cells contained within its cluster, and we excluded genes that were not expressed. This analysis revealed inter‐cluster differences in the frequency distributions of geographically localized transcriptional silencing events (Figure 2B). Clusters 4 and 8 (discrete to *Trf2* ^*−/−*^ cells) displayed a higher frequency of geographically clustered gene repression across somatic chromosomes relative to other clusters (Figure 2B). Moreover, when we compared the frequency of geographic silencing events involving 10 or more consecutive genes, *Trf2* ^*−/−*^ Clusters 4 and 8 exhibited a higher frequency of geographically clustered silencing across somatic chromosomes relative to similarly clustered *Trf2* ^*+/+*^ cells (Figure 2C). These data therefore suggest a higher burden of geographically clustered transcriptional repression in an inducible genetic model of senescence. Condensed DNA has previously been identified in senescent cells as so‐called senescence associated heterochromatic foci (SAHF) and there may be some overlap between the transcriptionally silent globules identified with MIMS (Narita et al. 2003). However, the geographical silencing implications have not previously been elucidated.

**FIGURE 2:**
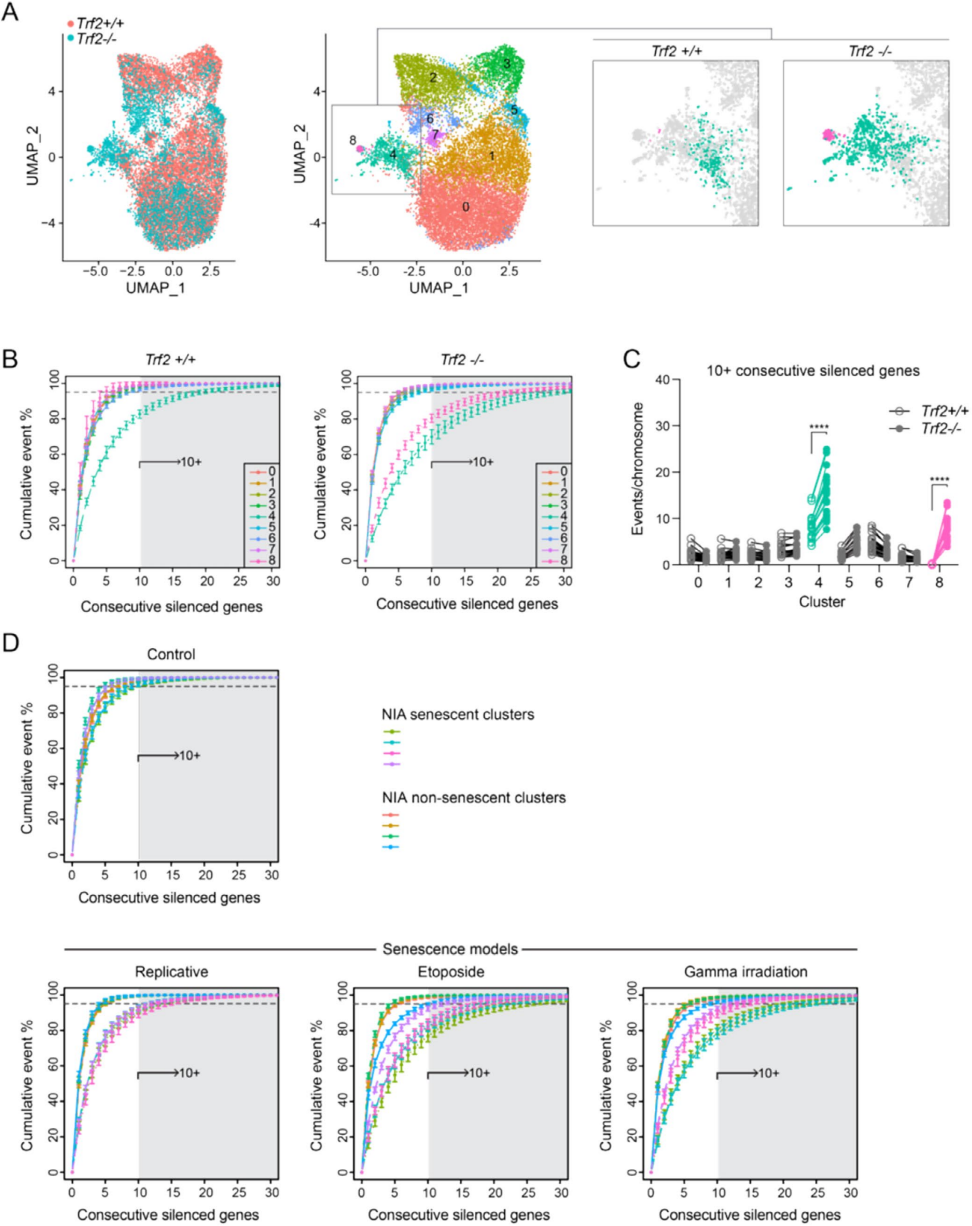
Cellular senescence is associated with transcriptional silencing in geographically clustered genes. (A) UMAP plots with emergence of distinct *Trf2* ^*−/−*^ cells, in particular Clusters 4 and 8 shown in insets. (B) Frequency of geographically clustered gene silencing in different UMAP clusters as a function of the number of consecutive genes meeting the silencing threshold (> 50% reduction in cluster‐specific transcript counts). (C) Frequency of 10+ consecutive silenced gene clusters for each somatic chromosome. ****p < 0.0001; two‐way ANOVA with Sidak's adjustment. (D) Frequency of geographically clustered gene silencing in NIA scRNA‐seq datasets, using same pipeline as in (B). Previously identified four senescent Clusters (2,4,6,7) (Wechter et al. 2023) exhibited rightward shift in the frequency of clustered transcriptional silencing events. In B and D, mean +/− s.d.m. for 19 somatic chromosomes.

We next tested for generalizability beyond the telomere uncapping model through re‐analysis of previously published scRNA‐seq for three established models of senescence in human diploid WI‐38 fibroblasts: replicative senescence, etoposide‐induced and gamma‐radiation‐induced senescence (Wechter et al. 2023). We applied the same pipeline as developed for the *Trf2* ^*−/−*^ model. With stress‐induced senescence, the geographic silencing effect was even more dramatic with putative senescent clusters showing a shift in the frequency of geographically clustered transcriptional repression (Figure 2D). We also examined whether specific functional categories were over‐represented amongst geographically suppressed genes, using prevalence in more than 20% of cells as threshold for inclusion. Gene set enrichment (GSEA) of these collective genes revealed Reactome pathways related to the cell cycle (Figure S5). In addition, the putative senescent clusters (*Trf2* Model 4, 8; Wechter et al. 2,4,6,7) displayed a high frequency of cells with loss of expression of high‐mobility group B (HMGB) family members, which has previously been shown to underlie the reorganization of chromatin structure associated with senescence (Sofiadis et al. 2021; Starkova et al. 2023) (Figure S6). Collectively, these sc‐RNAseq data provide orthogonal support for our MIMS observation of transcriptional silencing of large chromosomal segments of relevance to senescence.

We next examined gene silencing on a per‐cell basis, using 10 consecutive genes as threshold for geographical transcriptional repression. Most cells exhibited few silencing events (0–25), however, there was an extreme tail in the frequency distribution extending above 150 events/cell (Figure 3A). Each of the senescence models exhibited a greater frequency of cells meeting this high threshold of geographically concentrated transcriptional repression relative to respective control cells (Figure 3B,C). We hypothesized that if an extreme burden of geographical clustering is biologically significant, common cellular response programs might emerge by reclassifying cells based on their geographical clustering burden rather than their gene expression profiles. This approach could reveal patterns in cellular behavior that are not captured by traditional gene expression‐based classifications. Reactome pathway analysis revealed enrichment of genes involved in ‘*metabolism of RNA’* and ‘*metabolism of proteins’* in the subset of cells with a high burden of geographical silencing across each of the senescence models (Figure 3D), suggesting common cellular responses to the silencing phenomenon.

**FIGURE 3:**
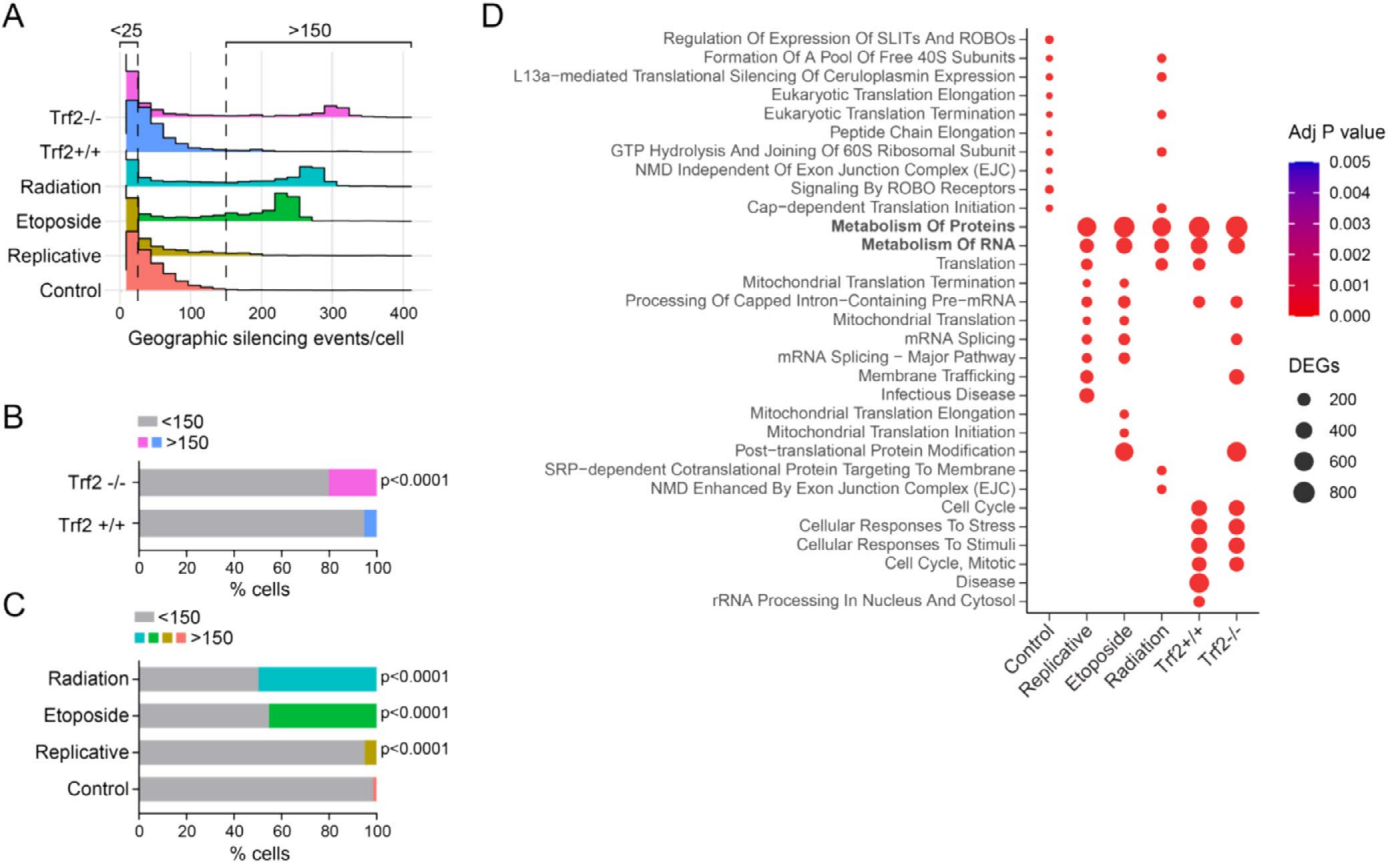
Common molecular responses in cells with high burden of geographical transcriptional silencing. (A) Histograms demonstrating extended tail in the frequency distributions of cells as a function of geographical silencing events using 10 consecutive genes as input. (B) Frequency of cells with high burden (> 150) silenced clusters in the genetic *Trf2* ^*−/−*^ senescence model. (C) Frequency of cells with high burden (> 150) silenced clusters in gamma‐radiation, etoposide, or replicative senescence models. Chi‐square *p* values relative to control shown for B and C. (D) GSEA analyses for Reactome gene sets, showing the top 10 enriched gene sets in cells with a high burden of geographical silencing (> 150) and compared to a cell population at the other extreme (< 25).

The fact that geographically clustered transcriptional silencing was evident in most clusters exposed to senescence‐inducing stressors (albeit in lower numbers) suggests that this state may define ‘early/pre‐senescence’. As for how large‐scale clustered transcriptional silencing may further result in deep senescence, we propose two possibilities. First, deep senescence arises when one or more critical combination of genes is stochastically sequestered in dense DNA globules. This model invokes repression of genes, rather than the activation of a specific gene program as an upstream mediator of senescence. This model is supported by the observation that the categories of genes, concentrated in chromosomal regions exhibiting putative geographical transcriptional silencing, included those related to the cell cycle (Figure S5) (Kandhaya‐Pillai et al. 2023). Second, the stochastic silencing of chromosomal segments in dense DNA globules, activates a cellular stress program that in turn drives senescence independent of the specific genes and chromosomal segments involved (similar to a monosomy model). This is consistent with our observation that cells exhibiting a high burden of geographical transcriptional suppression displayed modulation of transcriptional programs related to cellular stress responses. This may resemble the impaired proliferation and p53 pathway activation observed in cells with monosomies (Chunduri et al. 2021). Irrespective, the silencing of genes at the scale of large DNA globules beyond the precision that is typical of single gene cis‐regulatory mechanisms may explain why senescence is generally viewed as irreversible.

Cellular senescence is induced by diverse stress stimuli and clearly plays a role in multiple physiological and pathological conditions. However, we have yet to find a robust signature for senescence. The well‐known markers of senescent cells such as cyclin‐dependent kinase inhibitors (p16, p21), DNA damage response (γH2AX activation), senescence‐associated β‐galactosidase activity (SA‐β‐gal), SASP (IL6, GDF15), and apoptosis resistance (BCL‐2) are not unique to senescence, often overlapping with DNA damage response, quiescence or other cell fates. Furthermore, at a single cell level it has been challenging to identify a population of cells that exhibit all of these markers, suggesting significant heterogeneity of senescence. This is apparent by a number of prior studies that have attempted to compile marker gene sets enriched for senescence, such as the SenMayo and Fridman & Tainsky (Saul et al. 2022; Fridman and Tainsky 2008). Although valuable, these senescence marker lists have limited overlap between them‐ suggesting that finding senescence markers based on expression of individual genes may be challenging.

We utilized imaging mass spectrometry of stable isotope tracers for RNA synthesis and discovered dramatic silencing of nascent RNA synthesis in condensed globules of DNA in telomere uncapping genetic model of senescence. We then applied single‐cell transcriptomics in the telomere uncapping model, as well as in several additional models, to examine whether senescence‐associated clustered transcriptional repression occurs across these senescence models. This phenomenon was observed across all senescence models analyzed but was most pronounced in models that are considered more potent.

A limitation of our analyses, however, is that we did not directly define the DNA globules observed with MIMS at the individual gene or chromosomal level, nor did we identify globule associated proteins. Therefore, whether these condensed globules of DNA exhibit heterochromatin protein 1 (HP1) as seen with other examples of stereotypical architectural changes during senescence, such as senescence‐associated heterochromatin foci (SAHF), is unclear (Narita et al. 2003; Zhang et al. 2007). With improvements to correlative imaging and spatial transcriptomics, it may be possible in the future to characterize DNA globules identified by MIMS at the epigenetic and genetic levels, recognizing inherent challenges related to merging complementary methods with distinct sample preparations coupled with the inherently destructive nature of the MIMS ionization process at the sample surface. Future studies should also evaluate whether such large‐cluster gene silencing occurs in vivo under multiple stress conditions.

Nonetheless, our findings provide insight into how senescence‐inducing stimuli affect the large‐cluster gene expression landscape and offers a plausible rationale for both the molecular heterogeneity of senescence, as well as the challenge in defining universal molecular senescence markers, particularly activated genes. We propose that such large‐cluster gene silencing may act as both, a candidate driver and marker of the senescence state.

## Author Contributions

A.U.G. and M.L.S. provided study design. M.L.S., S.O., and A.U.G. acquired, analyzed, or interpreted data. C.G. and M.L.S. performed the MIMS study and analysis. K.C. and A.U.G. generated the senescent cells.

## Conflicts of Interest

The authors declare no conflicts of interest.

## Supporting information


Figure S1–6. Supplemental Figures and Methods

**Table S1.** GSEA identifies differentially expressed REACTOME pathways that are downregulated in (S1) Cluster 4 and (S2) Cluster 8 of tamoxifen treated (*Trf2* ^‐/‐^) versus control (*Trf2* ^+/+^).

## Data Availability

The data that support the findings of this study are openly available in GEO at https://www.ncbi.nlm.nih.gov/geo/query/acc.cgi?acc=GSE226225, reference number GEO (GSE226225).

## References

[acel70015-bib-0001] Alder, J. K. , C. E. Barkauskas , N. Limjunyawong , et al. 2015. “Telomere Dysfunction Causes Alveolar Stem Cell Failure.” Proceedings of the National Academy of Sciences of the United States of America 112, no. 16: 5099–5104. 10.1073/pnas.1504780112. 25840590 PMC4413294

[acel70015-bib-0002] Chunduri, N. K. , P. Menges , X. Zhang , et al. 2021. “Systems Approaches Identify the Consequences of Monosomy in Somatic Human Cells.” Nature Communications 12, no. 1: 5576. 10.1038/s41467-021-25288-x. PMC8458293 34552071

[acel70015-bib-0003] Fridman, A. L. , and M. A. Tainsky . 2008. “Critical Pathways in Cellular Senescence and Immortalization Revealed by Gene Expression Profiling.” Oncogene 27, no. 46: 5975–5987. 10.1038/onc.2008.213. 18711403 PMC3843241

[acel70015-bib-0004] Gorgoulis, V. , P. D. Adams , A. Alimonti , et al. 2019. “Cellular Senescence: Defining a Path Forward.” Cell 179, no. 4: 813–827. 10.1016/j.cell.2019.10.005. 31675495

[acel70015-bib-0005] Guillermier, C. , P. K. Fazeli , S. Kim , et al. 2017. “Imaging Mass Spectrometry Demonstrates Age‐Related Decline in Human Adipose Plasticity.” JCI Insight 2, no. 5: e90349. 10.1172/jci.insight.90349. 28289709 PMC5333969

[acel70015-bib-0006] Guillermier, C. , N. V. Kumar , R. C. Bracken , et al. 2024. “Nanoscale Imaging of DNA‐RNA Identifies Transcriptional Plasticity at Heterochromatin.” Life Science Alliance 7, no. 12: e202402849. 10.26508/lsa.202402849. 39288993 PMC11408601

[acel70015-bib-0007] Gurkar, A. U. , A. A. Gerencser , A. L. Mora , et al. 2023. “Spatial Mapping of Cellular Senescence: Emerging Challenges and Opportunities.” Nature Aging 3, no. 7: 776–790. 10.1038/s43587-023-00446-6. 37400722 PMC10505496

[acel70015-bib-0008] Gyngard, F. , and M. L. Steinhauser . 2019. “Biological Explorations With Nanoscale Secondary Ion Mass Spectrometry.” Journal of Analytical Atomic Spectrometry 34, no. 8: 1534–1545. 10.1039/C9JA00171A. 34054180 PMC8158666

[acel70015-bib-0009] Kandhaya‐Pillai, R. , F. Miro‐Mur , J. Alijotas‐Reig , et al. 2023. “Key Elements of Cellular Senescence Involve Transcriptional Repression of Mitotic and DNA Repair Genes Through the p53‐p16/RB‐E2F‐DREAM Complex.” Aging (Albany NY) 15, no. 10: 4012–4034. 10.18632/aging.204743. 37219418 PMC10258023

[acel70015-bib-0010] Narita, M. , S. Nũnez , E. Heard , et al. 2003. “Rb‐Mediated Heterochromatin Formation and Silencing of E2F Target Genes During Cellular Senescence.” Cell 113, no. 6: 703–716. 10.1016/s0092-8674(03)00401-x. 12809602

[acel70015-bib-0011] Saul, D. , R. L. Kosinsky , E. J. Atkinson , et al. 2022. “A New Gene Set Identifies Senescent Cells and Predicts Senescence‐Associated Pathways Across Tissues.” Nature Communications 13, no. 1: 4827. 10.1038/s41467-022-32552-1. PMC9381717 35974106

[acel70015-bib-0012] Sofiadis, K. , N. Josipovic , M. Nikolic , et al. 2021. “HMGB1 Coordinates SASP‐Related Chromatin Folding and RNA Homeostasis on the Path to Senescence.” Molecular Systems Biology 17, no. 6: e9760. 10.15252/msb.20209760. 34166567 PMC8224457

[acel70015-bib-0013] Starkova, T. , A. Polyanichko , A. N. Tomilin , and E. Chikhirzhina . 2023. “Structure and Functions of HMGB2 Protein.” International Journal of Molecular Sciences 24, no. 9: 8334. 10.3390/ijms24098334. 37176041 PMC10179549

[acel70015-bib-0014] Steinhauser, M. L. , A. P. Bailey , S. E. Senyo , et al. 2012. “Multi‐Isotope Imaging Mass Spectrometry Quantifies Stem Cell Division and Metabolism.” Nature 481, no. 7382: 516–519. 10.1038/nature10734. 22246326 PMC3267887

[acel70015-bib-0015] Suryadevara, V. , A. D. Hudgins , A. Rajesh , et al. 2024. “SenNet Recommendations for Detecting Senescent Cells in Different Tissues.” Nature Reviews Molecular Cell Biology 25, no. 12: 1001–1023. 10.1038/s41580-024-00738-8. 38831121 PMC11578798

[acel70015-bib-0016] Wechter, N. , M. Rossi , C. Anerillas , et al. 2023. “Single‐Cell Transcriptomic Analysis Uncovers Diverse and Dynamic Senescent Cell Populations.” Aging (Albany NY) 15, no. 8: 2824–2851. 10.18632/aging.204666. 37086265 PMC10188353

[acel70015-bib-0017] Zhang, R. , W. Chen , and P. D. Adams . 2007. “Molecular Dissection of Formation of Senescence‐Associated Heterochromatin Foci.” Molecular and Cellular Biology 27, no. 6: 2343–2358. 17242207 10.1128/MCB.02019-06 PMC1820509

