# Supplementary material for: Large‐Scale Clustered Transcriptional Silencing Associated With Cellular Senescence
Source: Aging Cell. 2025 Feb 19;24(4):e70015. doi: 10.1111/acel.70015 (PMC11984675; doi:10.1111/acel.70015)
Supplement: Supplementary file 1 — Figure S1–6. Supplemental Figures and Methods [file ACEL-24-e70015-s002.docx]

**Large-scale clustered transcriptional silencing associated with cellular senescence**

**Supplemental Figures 1**–**6**

**Supplemental Methods**


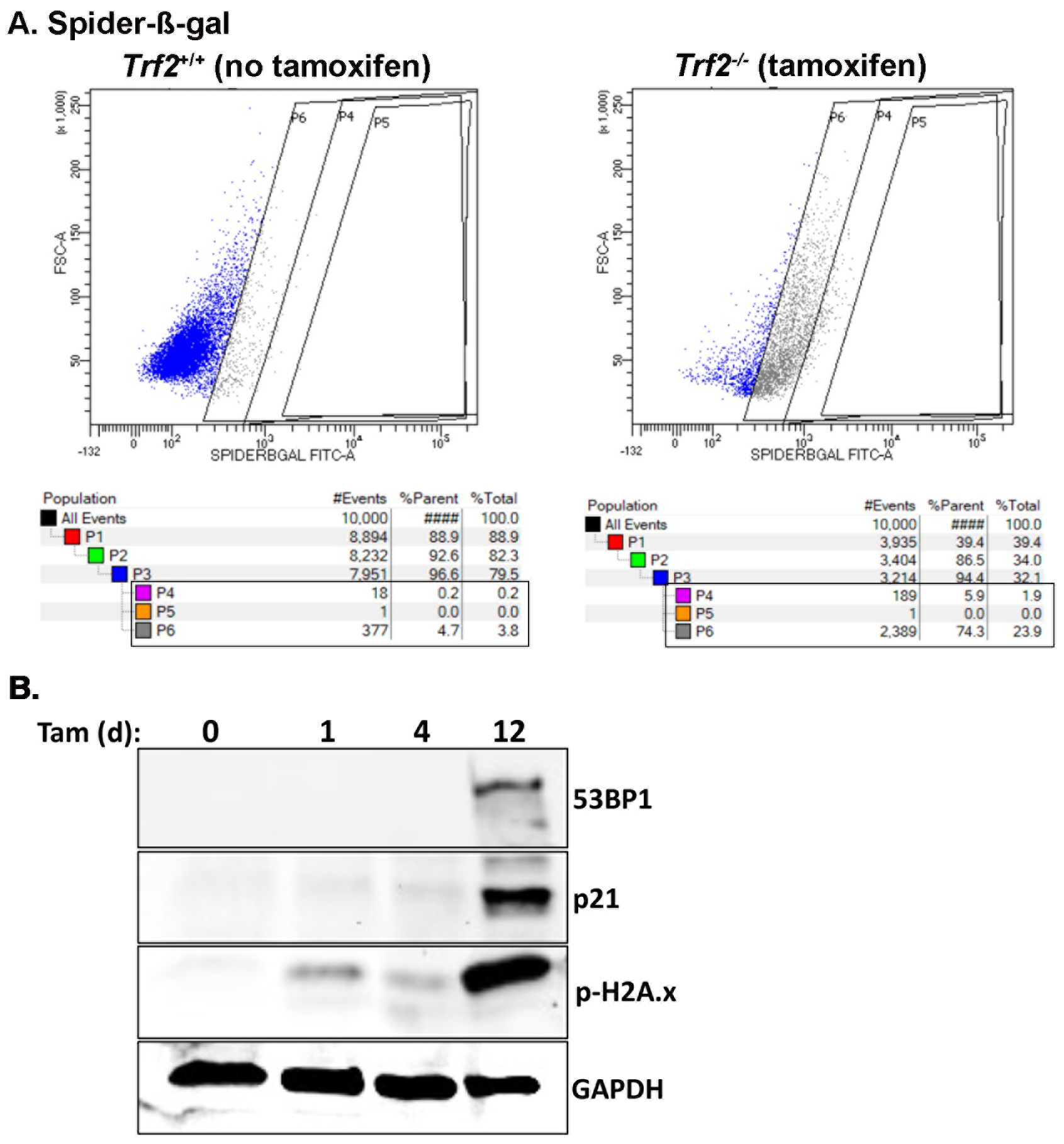


**Figure S1. Genetic senescence model**

**(A)** Flow cytometric analyses of SPiDERbGal in *Trf2^F/F^* *Rosa-CreERT 2* MEFs after tamoxifen treatment to induce Cre. Cells were treated with SPiDERbGal to assess senescence. ~23.9% cells are SPiDERbGal^high^ (gating P4-P6) in *Trf2^-/-^* cells. C.O.V. = 1.68%

**(B)** Induction of multiple DNA damage response genes and senescence markers in *Trf2^-/-^* cells, confirming previously established model (Alder et al., 2015).


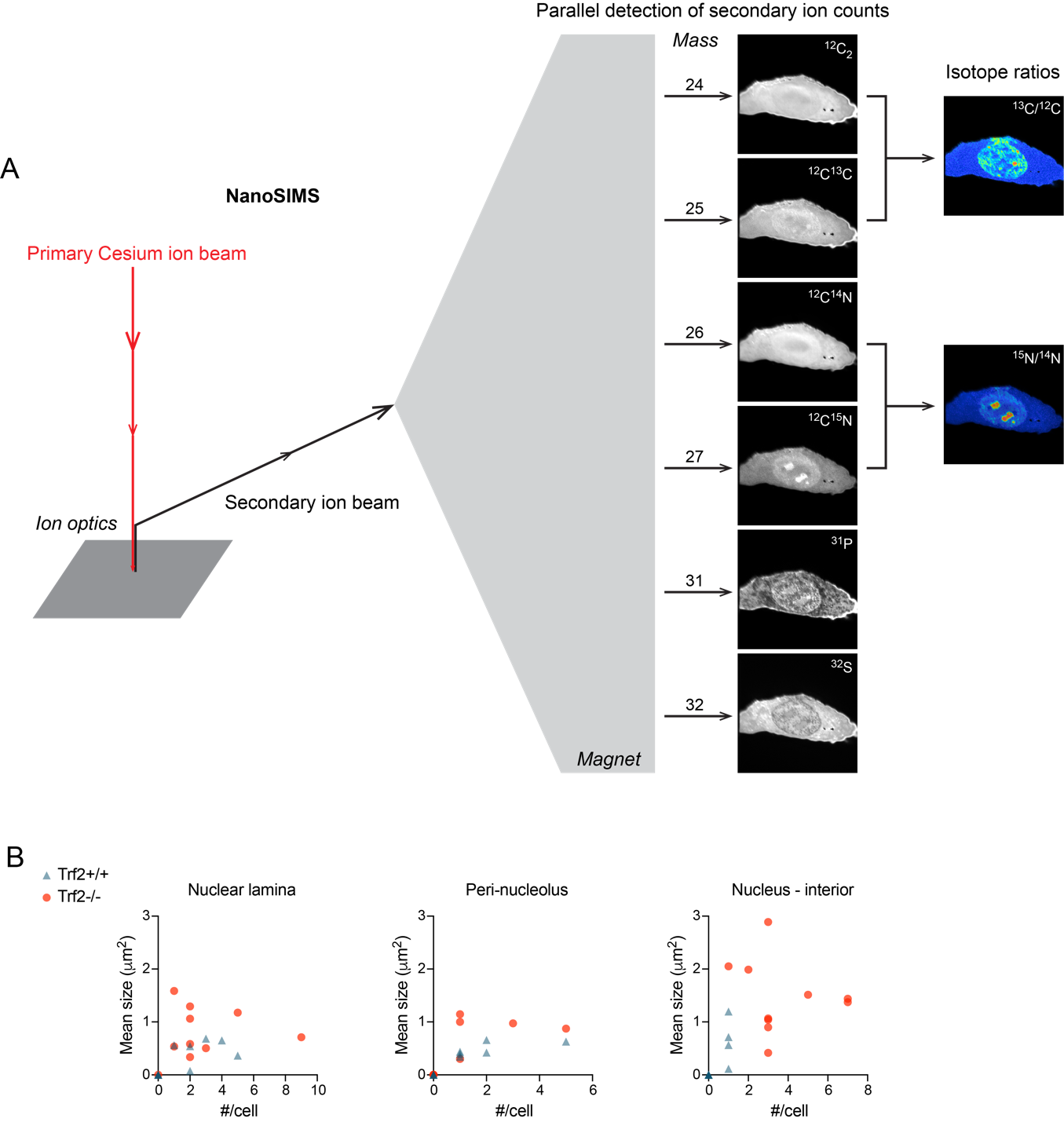


**Figure S2. Multi-isotope imaging mass spectrometry (MIMS) and discovery of nuclear remodeling in genetic senescence model.**

**(A)** MIMS merges stable isotope tracers with nanoscale secondary ion mass spectrometry (NanoSIMS). Ion optics shape coaxial ion beams. The primary Cesium beam sputters the sample surface liberating atoms and small polyatomic clusters (*e.g.* CN). Negatively charged ions ejected from the sputtered sample are shaped into a secondary ion beam which is separated by mass. Up to seven detectors are tuned to capture ionic species of interest. Note: each ion is measured in parallel from the same sputtered material. The instrument rasters the sample surface, with each measurement in turn forming the basis for a pixel in a quantitative mass image. This methodology was used to acquire data for Figures 1, S2, S3.

**(B)** Related to Figure 1B-E: punctate DNA dense and transcriptionally quiet regions were identified in control cells at the nuclear lamina and at the periphery of nucleoli, two nuclear regions where DNA typically condenses to form heterochromatin. Putative senescent cells exhibited similarly DNA dense and transcriptionally silent regions, but they were more numerous and with a larger globular character, particularly in the interior of the nucleus (non-nuclear lamina).


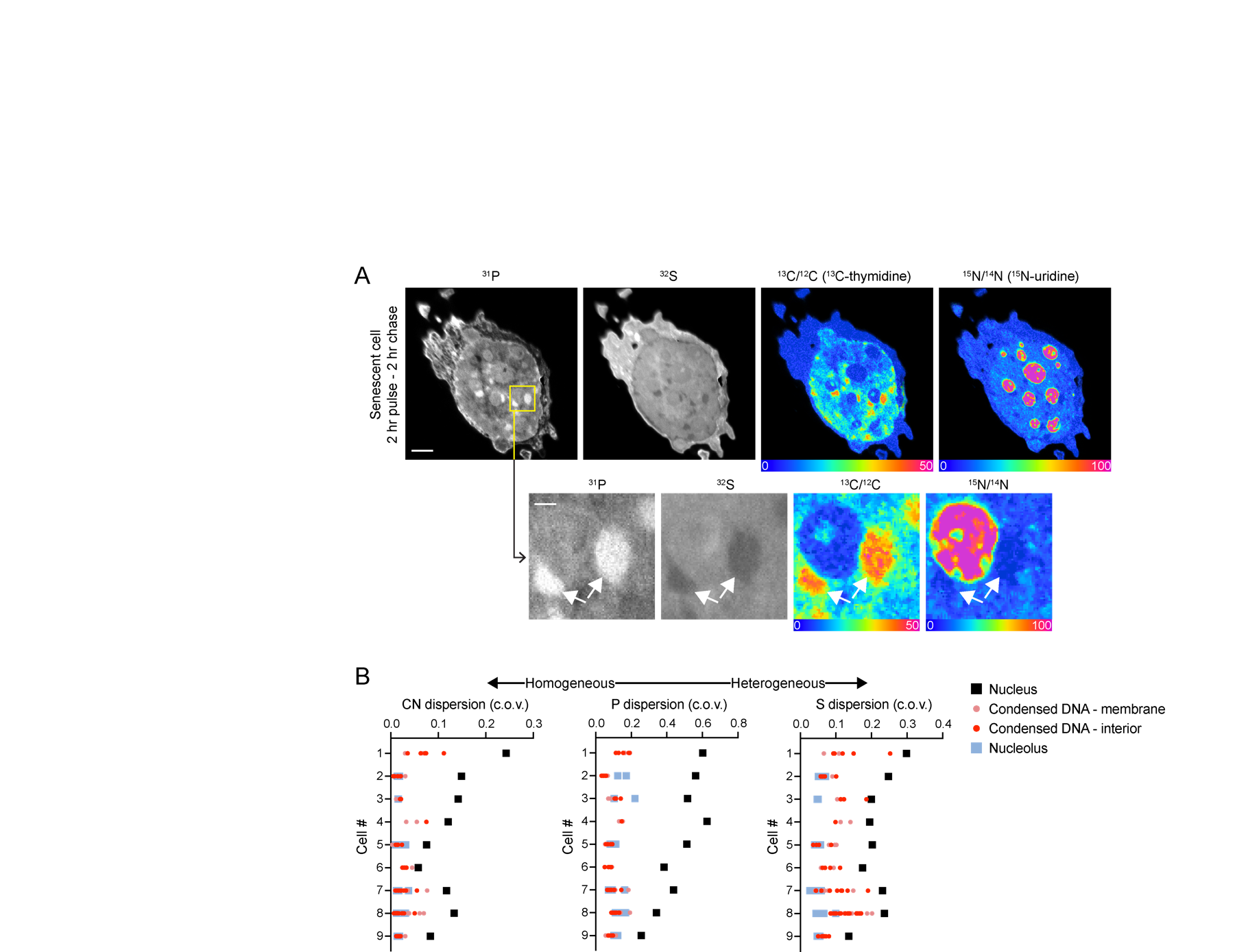


**Figure S3. Phase separation as a candidate mediator of senescent associated transcriptional repression in intranuclear DNA globules.**

**(A)** Representative *Trf2^-/-^* cell after 120 min ^15^N-uridine pulse and 120 min chase. High degree of ^15^N-uridine labeling of nucleoli persists with label free chase and dense DNA puncta remain devoid of nascent RNA. Despite high concentrations of nascent ^15^N-labeled RNA in nucleoli (arrows, inset), no evident transfer into adjacent DNA condensates was observed. Scale bar top row = 5 μm. Inset scale bar = 1 μm.

**(B)** Intranuclear domains may segregate due to liquid liquid phase separation, a homogeneous sequestration of molecules within any given phase. We tested the relative degrees of homogeneity of different intranuclear domains, represented in this analysis by a dimensionless metric: coefficient of variation (C.O.V.). We examined three quantitative variables with MIMS (CN^-^, P^-^, and S^-^). For each, a lower C.O.V. indicates greater homogeneity. Condensed DNA, whether located at the nuclear membrane or in intranuclear globules demonstrated homogeneity often in line with that observed in nucleoli a known phase separated structure.

­**
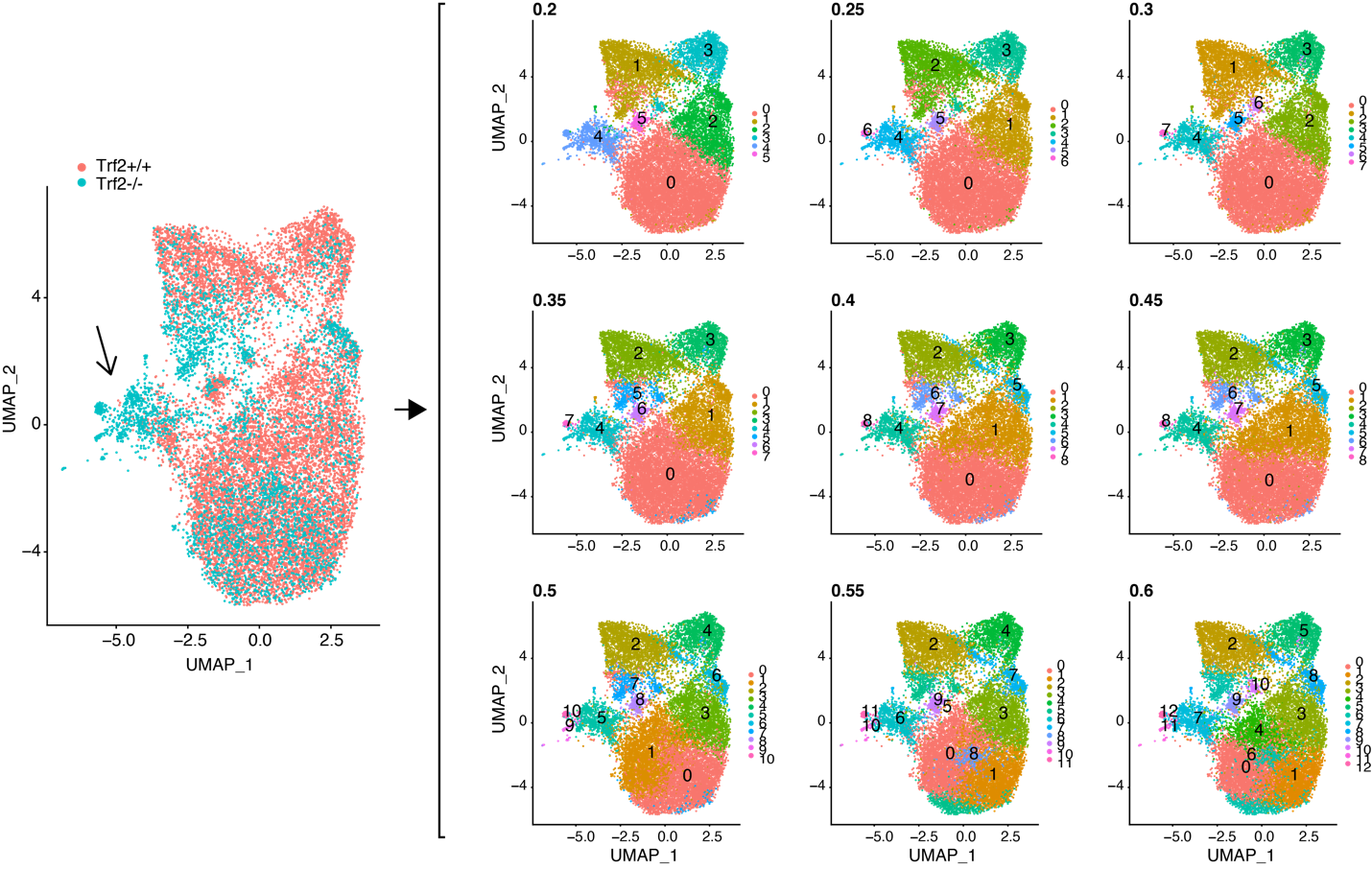
**

**Figure S4. Single cell RNA-sequencing and heterogeneity of Trf2 genetic model.**

UMAP plots were constructed at ascending levels of resolution ranging from 0.2-0.6. Distinct *Trf2^-/-^* cells emerged leftward in the UMAP plot (arrow). These cells were consistently distinct from the other clusters irrespective of resolution and grouped into 1-3 distinct clusters as a function of ascending levels of resolution. Analyses for this study were conducted at resolution=0.4. The significantly down-regulated gene sets in clusters 4, 8 (resolution 0.4) included pathways involved in cell cycle (Supplemental tables 1,2), consistent with a senescent phenotype.


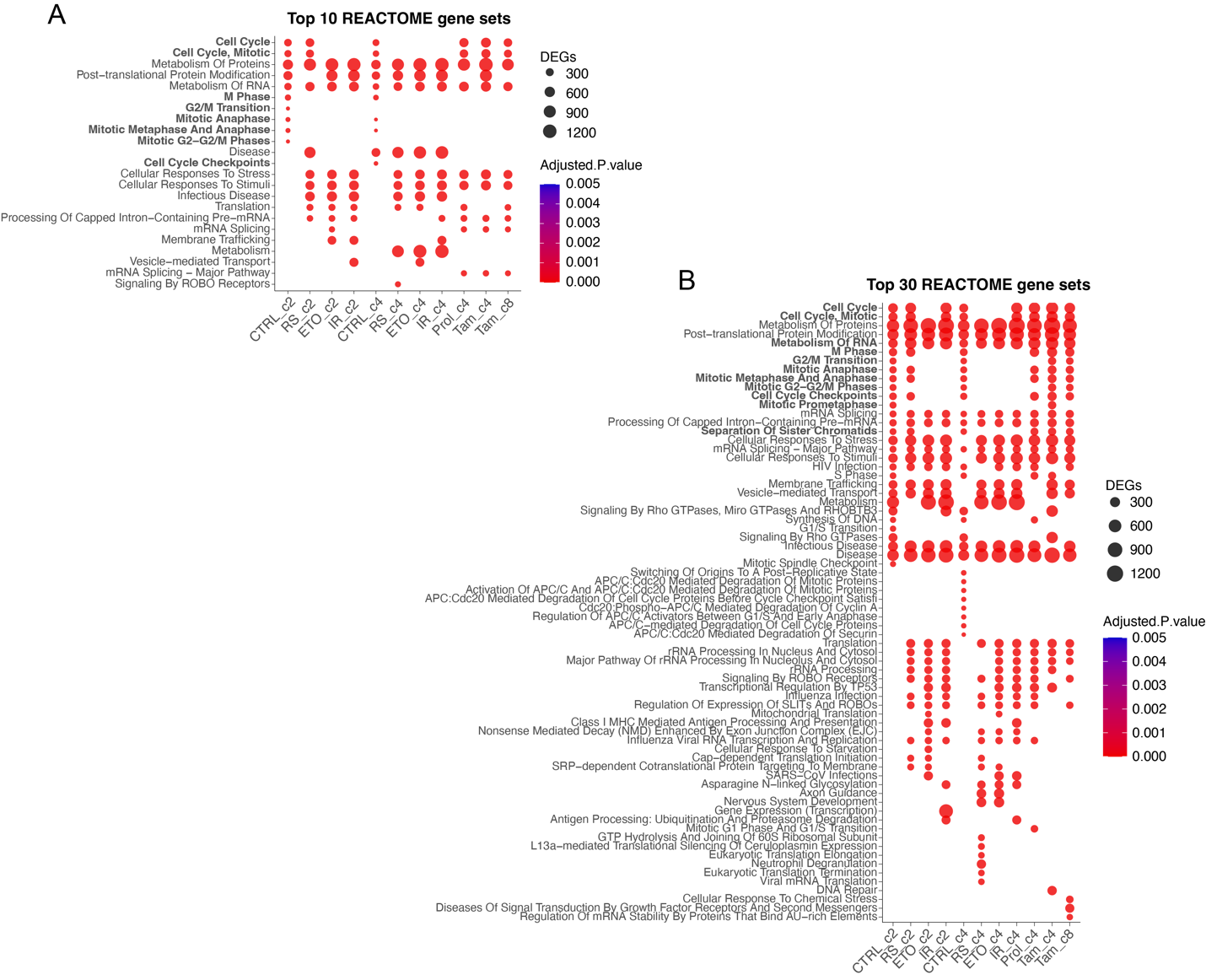


**Figure S5. Gene set enrichment analyses (GSEA) of genes found in geographically suppressed clusters.**

Criteria for inclusion as a high frequency suppressed gene were (i) part of a geographically suppressed gene cluster and (ii) present in at least 20% of the cells within a given senescent cell cluster (NIA clusters: 2, 4; Trf2 clusters 4, 8). GSEA analyses (REACTOME) then performed on the resultant genes.

**(A)** The top 10 enriched gene sets for each senescent cell group.

**(B**) The top 30 enriched gene sets for each senescent cell group.

Gene sets related to the cell cycle are bolded.

**
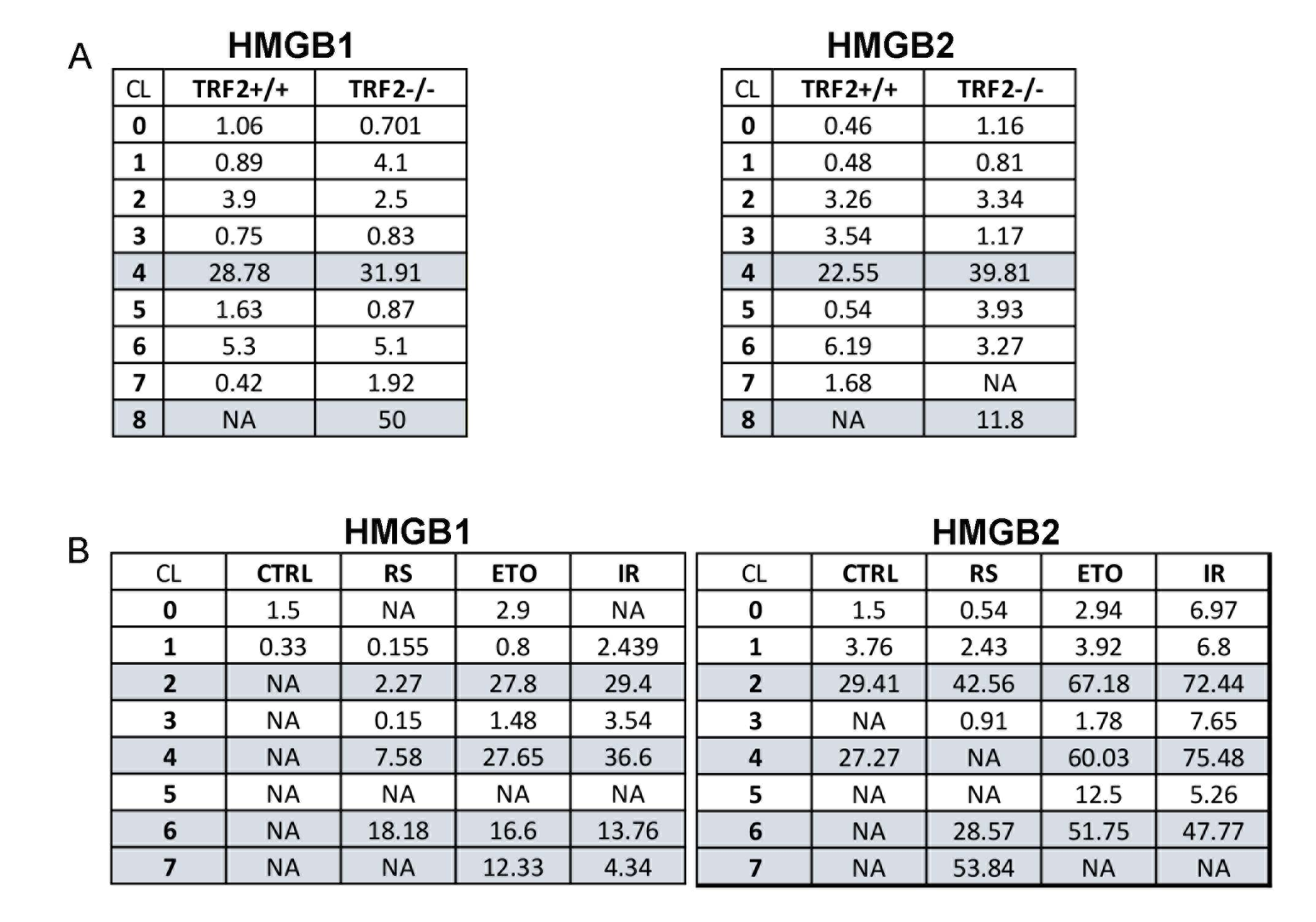
**

**Figure S6. HMGB1 and HMGB2 status**

Percent of HMGB1 and HMGB2 silenced cells in each cluster in **(A)** *Trf2^+/+^ (control)* and *Trf2^-/-^* telomere uncapping model of senescence in MEFs **(B)** WI38 untreated (CTRL= control); RS = replicative senescence; ETO = etoposide; and IR = gamma radiation. Blue highlighted clusters are putatively senescent

**Methods**

*Cell culture*

Trf2^F/F^ Rosa-CreERT 2 MEFs were cultured in DMEM supplemented with 10% fetal bovine serum and penicillin (120 U/mL), streptomycin (100 mcg/mL), and L-glutamine (2 mM). 4-hydroxytamoxifen (4HT/tamoxifen; 1 μM final concentration) was added at each passage and every 48 h. Cells were cultured for 12 days to ensure senescence. Treatment with tamoxifen results in *Tfr2* deletion, telomere uncapping and rapid and uniform senescence.

*Immunoblotting*

Cells were harvested on respective days after tamoxifen treatment by trypsinization and washed twice with phosphate-buffered saline (PBS). The cell pellets were lysed in RIPA buffer (Thermo Fisher Scientific) containing protease and phosphatase inhibitor cocktail (Sigma-Aldrich). Protein concentration was determined using the bicinchoninic acid (BCA) assay (Thermo Fisher Scientific) according to the manufacturer’s instructions. Equal amounts of protein (20–30 µg) were denatured by heating at 95°C for 5 minutes in Laemmli sample buffer (Bio-Rad) containing 5% β-mercaptoethanol. Proteins were separated on 10–12% SDS-polyacrylamide gels and transferred onto polyvinylidene difluoride (PVDF) membranes (Millipore) using a semi-dry transfer system. Membranes were blocked with 5% non-fat dry milk in Tris-buffered saline containing 0.1% Tween-20 (TBST) for 1 hour at room temperature. Membranes were incubated overnight at 4°C with primary antibodies against the following proteins: 53BP1 (1:1000, Cell Signaling Technology, Cat# 4937S); p21 (1:1000, Cell Signaling Technology, Cat# 2947S); H2AX (1:1000, Cell Signaling Technology, Cat# 7631S); GAPDH (1:5000, Abcam, Cat# 47724) used as a loading control. After washing three times with TBST, membranes were incubated with horseradish peroxidase (HRP)-conjugated secondary antibodies (1:10,000, Cell Signaling Technology) for 1 hour at room temperature. Protein bands were visualized using enhanced chemiluminescence (ECL) reagents (Thermo Fisher Scientific) and imaged with a chemiluminescence imaging system (Bio-Rad ChemiDoc).

*Senescence-Associated β-Galactosidase (SA-β-Gal) Staining and Analysis by Flow Cytometry*

Cells were harvested by trypsinization, washed with 1% BSA solution and were resuspended in 1X CellEvent™ Detection Reagent diluted in PBS. Staining was performed at 37°C for 2 hours in the dark to allow for substrate conversion by β-galactosidase. After incubation, cells were washed twice with 1% BSA to remove excess staining reagent and filtered through a 40 µm cell strainer to ensure a single-cell suspension. Flow cytometry was conducted using a Attune NXT (Thermo Fisher Scientific) flow cytometer. A minimum of 10,000 events per sample were acquired, and fluorescence intensity of the SA-β-Gal substrate was detected in the FITC channel. Forward and side scatter parameters were used to exclude debris and doublets. SA-β-Gal positive cells were gated based on fluorescence intensity above the threshold determined using an unstained control and Day 0 (baseline) samples.

*Multi-isotope imaging mass spectrometry*

Murine embryonic fibroblasts were grown in media containing ^13^C-thymidine (50 mM) similar to prior protocols (Steinhauser et al., 2012). Adherent cells were then seeded onto silicon wafers with the final passage prior to induction of *Trf2* recombination with tamoxifen. During senescence induction, cells were maintained in ^13^C-thymidine containing media prior to ^15^N-uridine (50 mM) pulse-chase labeling as previously used for labeling of nascent RNA (Bracken et al., 2024; Guillermier et al., 2024). Silicon wafers were fixed with 4% PFA and the cells dehydrated in ascending concentrations of ethanol. Samples were analyzed with a NanoSIMS 50L instrument (CAMECA).^13^C-thymidine labeling was measured by the ^13^C^12^C^–^/^12^C_2_^–^ ratio and ^15^N-uridine labeling by the ^12^C^15^N^–^/^12^C^14^N^–^ ratio as previously described (Bracken et al., 2024; Gyngard & Steinhauser, 2019; Kim et al., 2014; Steinhauser et al., 2012). The instrument was also tuned to generate ^31^P^-^ and ^32^S^-^ mass images used for delineation of intracellular structures, including DNA rich chromatin as previously described (Guillermier, Fazeli, et al., 2017). Image files were visualized and analyzed with a custom plugin to ImageJ: OpenMIMS 3.0: https://github.com/BWHCNI/OpenMIMS (Guillermier, Poczatek, Taylor, & Steinhauser, 2017). Mass images were used to guide manual selection of regions of interest (ROI) and the corresponding isotope ratios extracted from the pixels contained within each respective ROI. Isotope ratio data are displayed as hue saturation intensity (HSI) images. The lower bound of the scale (blue) was set at natural background (*e.g.,* for ^15^N-uridine data a lower bound of 0 is equivalent to the natural background of 0.37%=no labeling and an upper bound of 100 corresponds to a ratio of 0.74%). For images displayed in the figures, the upper bound of the scale was set to demonstrate regional differences in labeling, however the underlying quantitative data are unmodified by scaling changes to the images.

*scRNA-seq analysis*

Seurat v.4.0.1 R package was used for scRNA-seq analysis (Hao et al., 2021). Cells of low quality or doublets were excluded by filtering out cells with > 120000, < 1000 RNA counts, and/or with mitochondrial RNA percent > 15. Samples were integrated using the FindIntegrationAnchors and IntegrateData functions followed by SCTransform. Clustering was performed by the RunPCA, FindNeighbors with the first 30 principal components and FindClusters functions at resolution 0.4. The 2D projection of the clustering was carried out by the RunUMAP function. The dataset from Wechter *et. al.* was used as originally processed (Wechter et al., 2023).

*Identification of consecutively suppressed genes*

In each cell cluster, the mean expression of each gene was calculated. Then each gene in each cell was defined as “suppressed” if its expression value was below the 50% of the mean. If the mean was already 0, then that gene was not considered in subsequent analyses. The X and Y chromosomes were excluded from the analysis, as they are subject to sex chromosome inactivation. The numbers of 2, 3, 4… and so on suppressed genes that were neighboring each other in the chromosome coordinates were counted in each cell.

*Statistics*

Statistical analyses were performed using Prism 10 (Graphpad) using two tailed tests and with an alpha of 0.05. PCA analyses also utilized Prism 10, using CN, P, S ion counts and C and N ratio data is input. We have previously used median absolute deviation (MAD) to describe heterogeneity of MIMS datasets (Zhang et al., 2020), and similarly applied it in this study as a metric of relative intranuclear pixel heterogeneity. MAD represents the median of every single data point’s (i.e. pixel) absolute difference from the overall population median. MAD is an indicator of dispersion that incorporates all data points, can be applied to normal and non-normal distributions, and is resilient to outliers because all data points are weighted equally (Hampel, 1974).

*Differential expression and pathway enrichment analysis*

Differential gene expression analysis between cells with less than 25 ten or more consecutively suppressed gene events and cells with more than 150 ten or more consecutively suppressed gene events in each cluster was conducted using the FindMarkers function with min.pct being 0.1 and the adjusted p-value cutoff of 0.05. DEGs from all clusters were pooled for each treatment condition and GSEA was performed on these pooled DEG sets with the enrichR R package (Kuleshov et al., 2016) using the “GO_Reactome_2022” database.

**Supplemental References**

Alder, J. K., Barkauskas, C. E., Limjunyawong, N., Stanley, S. E., Kembou, F., Tuder, R. M., . . . Armanios, M. (2015). Telomere dysfunction causes alveolar stem cell failure. *Proc Natl Acad Sci U S A, 112*(16), 5099-5104. doi:10.1073/pnas.1504780112

Bracken, R. C., Davison, L. M., Buehler, D. P., Fulton, M. E., Carson, E. E., Sheng, Q., . . . Brown, J. D. (2024). Transcriptional synergy in human aortic endothelial cells is vulnerable to combination p300/CBP and BET bromodomain inhibition. *iScience, 27*(6), 110011. doi:10.1016/j.isci.2024.110011

Guillermier, C., Fazeli, P. K., Kim, S., Lun, M., Zuflacht, J. P., Milian, J., . . . Steinhauser, M. L. (2017). Imaging mass spectrometry demonstrates age-related decline in human adipose plasticity. *JCI Insight, 2*(5), e90349. doi:10.1172/jci.insight.90349

Guillermier, C., Kumar, N. V., Bracken, R. C., Alvarez, D., O'Keefe, J., Gurkar, A., . . . Steinhauser, M. L. (2024). Nanoscale imaging of DNA-RNA identifies transcriptional plasticity at heterochromatin. *Life Sci Alliance, 7*(12). doi:10.26508/lsa.202402849

Guillermier, C., Poczatek, J. C., Taylor, W. R., & Steinhauser, M. L. (2017). Quantitative imaging of deuterated metabolic tracers in biological tissues with nanoscale secondary ion mass spectrometry. *Int J Mass Spectrom, 422*, 42-50. doi:10.1016/j.ijms.2017.08.004

Gyngard, F., & Steinhauser, M. L. (2019). Biological explorations with nanoscale secondary ion mass spectrometry. *Journal of Analytical Atomic Spectrometry, 34*(8), 1534-1545. doi:10.1039/C9JA00171A

Hampel, F. R. (1974). The Influence Curve and its Role in Robust Estimation. *Journal of the American Statistical Association, 69*(346), 383-393. doi:10.1080/01621459.1974.10482962

Hao, Y., Hao, S., Andersen-Nissen, E., Mauck, W. M., Zheng, S., Butler, A., . . . Satija, R. (2021). Integrated analysis of multimodal single-cell data. *Cell, 184*(13), 3573-3587.e3529. doi:10.1016/j.cell.2021.04.048

Kim, S. M., Lun, M., Wang, M., Senyo, S. E., Guillermier, C., Patwari, P., & Steinhauser, M. L. (2014). Loss of white adipose hyperplastic potential is associated with enhanced susceptibility to insulin resistance. *Cell Metab, 20*(6), 1049-1058. doi:10.1016/j.cmet.2014.10.010

Kuleshov, M. V., Jones, M. R., Rouillard, A. D., Fernandez, N. F., Duan, Q., Wang, Z., . . . Ma'ayan, A. (2016). Enrichr: a comprehensive gene set enrichment analysis web server 2016 update. *Nucleic Acids Res, 44*(W1), W90-97. doi:10.1093/nar/gkw377

Meigs, J. B., Cupples, L. A., & Wilson, P. W. (2000). Parental transmission of type 2 diabetes: the Framingham Offspring Study. *Diabetes, 49*(12), 2201-2207. doi:10.2337/diabetes.49.12.2201

Steinhauser, M. L., Bailey, A. P., Senyo, S. E., Guillermier, C., Perlstein, T. S., Gould, A. P., . . . Lechene, C. P. (2012). Multi-isotope imaging mass spectrometry quantifies stem cell division and metabolism. *Nature, 481*(7382), 516-519. doi:nature10734 [pii]10.1038/nature10734

Wechter, N., Rossi, M., Anerillas, C., Tsitsipatis, D., Piao, Y., Fan, J., . . . Gorospe, M. (2023). Single-cell transcriptomic analysis uncovers diverse and dynamic senescent cell populations. *Aging (Albany NY), 15*(8), 2824-2851. doi:10.18632/aging.204666

Zhang, Y., Guillermier, C., De Raedt, T., Cox, A. G., Maertens, O., Yimlamai, D., . . . Steinhauser, M. L. (2020). Imaging Mass Spectrometry Reveals Tumor Metabolic Heterogeneity. *iScience, 23*(8), 101355. doi:10.1016/j.isci.2020.101355
